# Ethanol Exposure to Ethanol-Oxidizing HEPG2 Cells Induces Intracellular Protein Aggregation

**DOI:** 10.3390/cells12071013

**Published:** 2023-03-26

**Authors:** Paul G. Thomes, Gage Rensch, Carol A. Casey, Terrence M. Donohue

**Affiliations:** 1 Liver Study Unit, VA-Nebraska-Western Iowa Health Care System, Omaha, NE 68105, USA; 2 The Depts of Internal Medicine, University of Nebraska Medical Center, Omaha, NE 68198, USA; 3 Biochemistry/Molecular Biology, University of Nebraska Medical Center, Omaha, NE 68198, USA

**Keywords:** EtOH, acetaldehyde, aggresome, proteasome, autophagy

## Abstract

Background: Aggresomes are collections of intracellular protein aggregates. In liver cells of patients with alcoholic hepatitis, aggresomes appear histologically as cellular inclusions known as Mallory–Denk (M–D) bodies. The proteasome is a multicatalytic intracellular protease that catalyzes the degradation of both normal (native) and abnormal (misfolded and/or damaged) proteins. The enzyme minimizes intracellular protein aggregate formation by rapidly degrading abnormal proteins before they form aggregates. When proteasome activity is blocked, either by specific inhibitors or by intracellular oxidants (e.g., peroxynitrite, acetaldehyde), aggresome formation is enhanced. Here, we sought to verify whether inhibition of proteasome activity by ethanol exposure enhances protein aggregate formation in VL-17A cells, which are recombinant, ethanol-oxidizing HepG2 cells that express both alcohol dehydrogenase (ADH) and cytochrome P450 2E1 (CYP2E1). Methods: We exposed ethanol-non-oxidizing HepG2 cells (^ADH−/CYP2E1−^) or ethanol-oxidizing VL-17A (^ADH+/CYP2E1+^) to varying levels of ethanol for 24 h or 72 h. After these treatments, we stained cells for aggresomes (detected microscopically) and quantified their numbers and sizes. We also conducted flow cytometric analyses to confirm our microscopic findings. Additionally, aggresome content in liver cells of patients with alcohol-induced hepatitis was quantified. Results: After we exposed VL-17A cells to increasing doses of ethanol for 24 h or 72 h, 20S proteasome activity declined in response to rising ethanol concentrations. After 24 h of ethanol exposure, aggresome numbers in VL-17A cells were 1.8-fold higher than their untreated controls at all ethanol concentrations employed. After 72 h of ethanol exposure, mean aggresome numbers were 2.5-fold higher than unexposed control cells. The mean aggregate size in all ethanol-exposed VL-17A cells was significantly higher than in unexposed control cells but was unaffected by the duration of ethanol exposure. Co-exposure of cells to EtOH and rapamycin, the latter an autophagy activator, completely prevented EtOH-induced aggresome formation. In the livers of patients with alcohol-induced hepatitis (AH), the staining intensity of aggresomes was 2.2-fold higher than in the livers of patients without alcohol use disorder (AUD). Conclusions: We conclude that ethanol-induced proteasome inhibition in ethanol-metabolizing VL-17A hepatoma cells causes accumulation of protein aggregates. Notably, autophagy activation removes such aggregates. The significance of these findings is discussed.

## 1. Introduction

Conformational diseases are characterized by excessive intracellular accumulation of misfolded, entangled proteins known as aggregates. Such aggregates are sequestered into distinct pericentriolar compartments called aggresomes. It is generally believed that aggresome formation is cytoprotective—part of a distinct cellular response that limits the spread of misfolded proteins [1]. Protein misfolding has a number of causes: Missense mutations cause amino acid substitutions in nascent proteins, thereby slowing or preventing normal protein folding during de novo protein synthesis [2,3]. Protein misfolding also occurs by adduct formation, when mature proteins covalently bind to reactive metabolites, including acetaldehyde (Ach), malondialdehyde (MDA), or 4-hydroxynonenal (4-HNE). Once formed, the aforementioned adducts alter proteins’ biological activities by causing their denaturation and aggregation, the latter through the formation of intramolecular and intermolecular cross-links [4,5,6,7]. Similarly, reactions of proteins with short-lived reactive oxygen species (ROS), including superoxide (O^−^_2_), hydrogen peroxide (H_2_O_2_), hydroxyl radicals (_●_OH), or peroxynitrite (HONOO^−^), initiate oxidation of protein-bound thiols or form covalent adducts with tyrosyl residues to form 3-nitrotyrosine [8].

To minimize aggregation, nascent proteins that carry missense mutations are recognized as misfolded ribosome-bound growing polypeptides that are degraded by the ubiquitin-proteasome system (UPS) [9] before they become entangled into insoluble aggregates. Other completed proteins, after they are modified by reactive metabolites, are selectively degraded by the 20S proteasome, which rather selectively recognizes damaged or modified proteins [5,10,11]. However, if proteasome activity declines, misfolded proteins evade proteolysis, forming proteasome-resistant insoluble oligomers that can only be degraded by autophagy. The latter is a crucial hydrolytic pathway by which all macromolecular forms are broken down in lysosomes [12,13].

Excessive alcohol consumption causes hepatic protein accumulation (proteopathy) and fatty liver (steatosis). Both of these contribute to alcohol-induced hepatomegaly (liver enlargement), which is common in problem drinkers. Proteopathy is associated with the formation of Mallory–Denk (M–D) bodies, the histological signatures of alcohol-induced liver disease [14,15,16]. M–D bodies are composed of aggregates of filamentous, cytokeratin-rich, ubiquitylated, misfolded proteins that accumulate because they are resistant to proteasome-catalyzed degradation [17]. When M–D bodies are chemically induced in livers of mice, some hepatocytes are devoid of a catalytically essential proteasome subunit, implying that reduced proteasome activity exacerbates M–D body formation [18]. Further work has revealed that proteasome activity significantly declines after EtOH exposure to recombinant Hep G2 (E-47) cells that constitutively overexpress CYP2E1. The EtOH-induced decline in proteasome activity is inversely related to accumulation of cytokeratin-rich aggresomes, confirming that proteasome inhibition promotes aggregate/aggresome development [19]. Later investigation revealed that prolonged exposure of E-47 cells to EtOH inhibits autophagy, implying that such inhibition likely intensifies aggresome accumulation [20].

Here, we sought to determine whether proteasome inhibition by ethanol exposure is associated with protein aggregate formation in ethanol-metabolizing VL-17A cells that constitutively express alcohol dehydrogenase (ADH) and cytochome P450 2E1 (CYP2E1). Our findings indicated that ethanol treatment increases cellular aggresome formation, which requires the oxidation of ethanol to acetaldehyde.

## 2. Materials and Methods

### 2.1. Animal Treatments

All animal protocols were approved by the Institutional Animal Care and Use Committee at the Research Service of the Department of Veterans’ Affairs Nebraska–Western Iowa Health Care System. We followed the eighth edition of the *Guide for the Care and Use of Laboratory Animals*, published by the National Institutes of Health. Male Wistar rats weighing 175–200 g and purchased from Charles River Laboratories (Portage, MI, USA) were weight-matched and fed control or EtOH-containing Lieber-DeCarli liquid diets for 6 wks [21]. When rats were sacrificed, after pair-feeding control or EtOH diets, we isolated hepatocytes from both groups of animals as previously described [22] and incubated the cells overnight (16 h) before treating and analyzing them, as described in the figure legends.

### 2.2. Cells and Treatments

We obtained HepG2 (human hepatoma) cells (^ADH−/CYP2E1−^) from the American Type Culture Collection (ATCC). VL-17A cells (^ADH+/CYP2E1+)^ were seeded and grown as we previously described [23]. When we grew VL-17A cells, we added the selective antibiotics zeocin and G-418 sulfate (each at 400 μg/mL) to the medium to maintain expressions of ADH and CYP2E1, respectively [23]. However, during experiments, we excluded the selective antibiotics from the medium. We exposed cells to increasing concentrations of ethanol (zero to 100 mM), to the proteasome inhibitor MG132 (2.5 µM), or to the autophagy inducer rapamycin (100 nM) for the durations indicated in the figure legends.

### 2.3. Human Samples

We obtained de-identified paraffin-embedded sections (slides) of normal and alcoholic hepatitis (AH) livers from Dr. Zhaoli Sun, Director of Transplant Biology Research Center at the Johns Hopkins University School of Medicine. The Human sample repository at Johns Hopkins is supported by the NIH award R24AA025017.

### 2.4. Lactate Dehydrogenase Assay

The toxicities of treatments to VL17A cells were assessed by the release (leakage) of cellular lactate dehydrogenase (LDH) into the incubation media. LDH activity in media samples was measured spectrophotometrically, as published in [23].

### 2.5. Aggresome Detection

Using multi-well plates containing glass cover slips, we plated and grew VL-17A ^ADH+/CYP2E1+^ and HepG2 ^ADH−/CYP2E1−^ cells in DMEM, as just described. After exposing the cells to EtOH and/or other agents, we permeabilized and then stained the cells with Proteostat^®^ aggresome detection dye (Enzo, Inc., Farmingdale, NY, USA), using the manufacturer’s instructions. Flow cytometric detection of aggresomes was performed on intact cells incubated with aggresome detection dye according to the manufacturer’s instructions. Microscope images were captured with a digital confocal microscope. We quantified the numbers and sizes of protein aggregates in cell images with NIH Image J software. Data were normalized per cell nucleus. In human samples, we quantified the fluorescence intensities of aggresomes, as those aggregates in AH livers were large and were not individual puncta as seen in normal livers. We used 2 random images from 4 different normal and 4 different AH livers for aggresome quantification.

### 2.6. Proteasome Assay

We measured the chymotrypsin-like activity of the proteasome after incubating cell lysates with the fluorogenic peptide substrate, N-Suc-LLVY-AMC (Sigma Chemical, St. Louis, MO, USA), according to our published procedure [24]. One proteasome activity unit catalyzes the release of one nanomole of free AMC per hour. To minimize inter-experimental variation in proteasome specific activity, we express all proteasome specific activity data (units per mg protein) as the percent of the untreated control value from each experiment.

### 2.7. Reactive Oxygen Species (ROS) Quantification

Following their treatment with ethanol or acetaldehyde, we incubated VL-17A cells with the redox-sensitive fluorescent probe 2,7-Dichlorodihydrofluroscine diacetate (DFCDA). The fluorescence that resulted from oxidation of DFCDA by ROS was measured using a fluorescence plate reader, and the fluorescence was normalized to the protein concentration.

### 2.8. Statistical Analyses

Data are expressed as mean values ± standard error (S.E). We determined statistical significance between two groups by Student’s t-test and among multiple groups by one-way analysis of variance (ANOVA) using a Neuman–Keuls post hoc analysis. A probability (*p*) value ≤ 0.05 was considered statistically significant.

## 3. Results

### 3.1. Aggresome Content in Livers of Human Subjects with Alcohol-Induced Hepatitis (AH) Was Higher than in Normal Subjects

**Figure 1:**
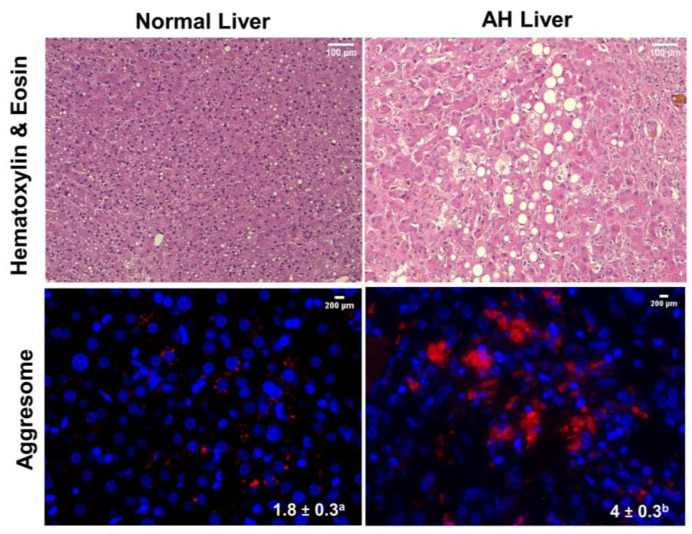
Hematoxylin and eosin (H&E) staining and aggresomes in normal human livers and in livers from patients with alcoholic hepatitis (AH). H&E staining is a representative image from one normal and one AH liver section. Quantification of aggresome staining intensity (red) is from 2 random images from each of 4 normal and 4 AH liver sections. Data are mean values ± SE. Superscripts with different letters indicate statistically significant differences between groups. (*p* ≤ 0.05).

To ascertain whether aggresome formation is a histopathological outcome in heavy drinkers, we used aggressome detection dye to stain liver sections from normal subjects and from those with alcoholic hepatitis (AH). The mean fluorescent staining intensity of aggresomes detected was 2.2-fold higher in AH livers than in normal livers (Figure 1). Hematoxylin and eosin (H&E) staining revealed that AH livers exhibited ballooned (swollen) hepatocytes with fat accumulation (macrovesicular steatosis) (Figure 1). Clinical data associated with normal and AH samples are presented in the Supplementary Materials, Table S1.

### 3.2. EtOH Exposure to VL-17A Cells Decreased Proteasome Activity and Enhanced Aggresome Detection

**Figure 2:**
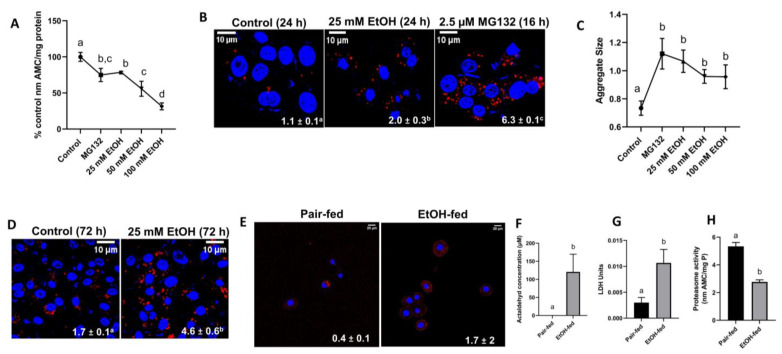
(**A**) Proteasome activity, (**B**,**D**) protein aggregates, and (**C**) aggregsome size in VL-17A cells exposed to MG132 and/or varying doses of ethanol for the times indicated in the abscissa. (**E**) protein aggregates, (**F**) media acetaldehyde levels (**G**), LDH leakage in incubation media, and (**H**) proteasome activity in hepatocytes isolated from EtOH-fed rats and their pair-fed controls and incubated overnight. On quantification shown at the bottom of micrographs or on line or bar graphs, the same letters (superscripts) are not significantly different. These data containing different letters (superscripts) are significantly different.

After a 24 h exposure of VL-17A cells to either 25 mM ethanol or a 16 h (overnight) exposure to 2.5 µM MG132, a potent proteasome inhibitor, we observed a 20–22% loss of proteasome chymotrypsin-like activity compared with unexposed control cells (Figure 2A). After we exposed VL-17A cells to higher (50 mM and 100 mM) ethanol concentrations for 24 h, there was a 45% and 70% decline in proteasome specific activity, respectively, compared with unexposed cells (Figure 2A). Such losses in proteasome activity were similar, even after 72 h of exposure to the aforementioned ethanol concentrations. VL-17A cells exposed to 25 mM ethanol for 24 h exhibited a 1.8-fold rise in aggregate/aggresome numbers (Figure 2B) and a 1.4-fold elevation in aggregate/aggresome size (Figure 2C) (i.e., the average area of an individual punctum) compared with unexposed cells. Cells exposed overnight to 2.5 μM MG132 contained nearly 6-fold higher aggresome numbers (Figure 2B) and 1.5-fold larger aggregate size (Figure 2C) than untreated cells. Aggresome numbers in MG132-treated VL-17A cells were 3-fold higher than in cells exposed 24 h to 25 mM ethanol (Figure 2B). However, the mean aggresome size in MG-132 treated cells was essentially the same as that in EtOH-exposed cells (Figure 2C). There were no differences in aggresome numbers after 24 h of exposure to 25, 50, or 100 mM EtOH. We observed similar results after 72 h of exposure to 25 mM, 50 mM, and 100 mM EtOH after which VL-17A cells exhibited 2.4-fold higher aggregate/aggresome numbers than unexposed cells (Figure 2D), but there were no EtOH dose-dependent changes in aggresome sizes. It is noteworthy that when the duration of incubation was extended to 72 h, unexposed control cells developed 54% higher numbers of aggregates than unexposed cells incubated for 24 h. We also detected numerically higher aggresome numbers in hepatocytes of EtOH-fed rats than in hepatocytes from pair-fed control rats (Figure 2E). Additionally, compared with hepatocytes from pair-fed controls, hepatocytes from EtOH-fed rats produced higher levels of extracellular acetaldehyde (Figure 2F) and they leaked more lactate dehydrogenase (LDH) activity into the extracellular medium (Figure 2G). Freshly isolated hepatocytes from EtOH-fed rats had half the proteasome specific activity than cells from pair-fed control rats (Figure 2H).

### 3.3. Acetaldehyde (Ach) Generation Promoted Aggresome Formation and Cytotoxicity in VL-17A Cells

**Figure 3:**
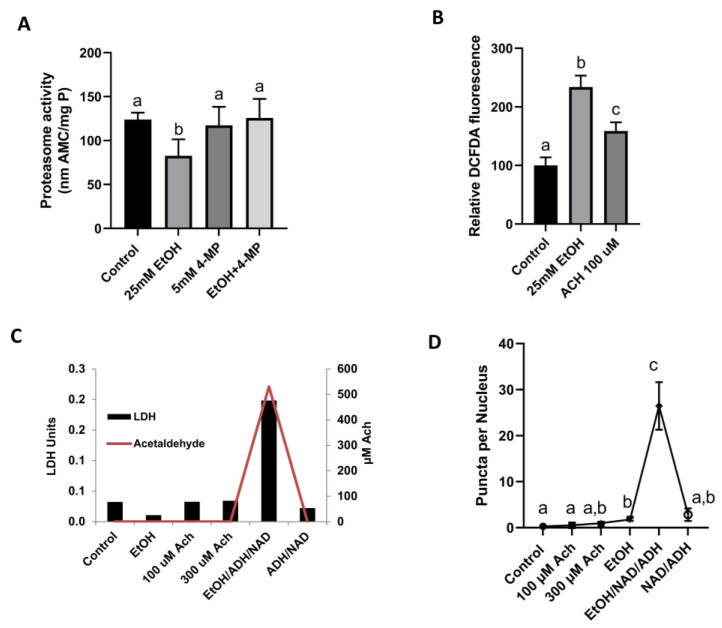
(**A**) Proteasome activity in VL-17A cells treated with 25 mM ethanol for 24 h in the presence and absence of 5 mM 4-methyl pyrazole (4-MP). (**B**) DCFDA fluorescence in VL-17A cell treated with 25 mM ethanol or 100 µM acetaldehyde for 30 min. (**C**) Cellular necrosis (LDH leakage) and Ach levels in HepG2 cells exposed 12 h to the indicated treatments on the *x*-axis. (**D**) aggresome puncta numbers in HepG2 cells exposed to the indicated treatments. Letter symbols mean the same as in Figure 1 and Figure 2. N = 5–10 microscope fields per group.

Figure 2 shows that the degree of proteasome activity decline in EtOH-exposed VL-17A cells depended on the initial EtOH concentration. These data suggest that the EtOH-elicited inhibition of the proteasome likely depends on the degree of EtOH oxidation to acetaldehyde (Ach) in these cells, which generated up to 300 µM Ach in the culture medium after exposure to 50 mM EtOH. To confirm this, we co-incubated ethanol exposed cells with 4-methylpyrazol (4-MP) which inhibits ADH activity to prevent acetaldehyde production. Co-incubation of VL-17A cells with 5 mM 4-MP along with 25 mM ethanol for 24 h completely blocked ethanol-induced proteasome decline (Figure 2A). To determine if reactive oxygen species (ROS) and reactive nitrogen species (RNS) produced during ethanol metabolism in VL-17A cells also contribute to downregulation of proteasome activity, we measured fluorescence levels of DCFDA, a redox sensitive fluorescent probe that produces fluorescence following its oxidation by ROS and RNS. Incubation of VL-17A cells with DCFDA dye after their treatment with 0 mM or 25 mM ethanol for 3 h or 24 h did not produce significant difference in fluorescence levels between ethanol-treated and untreated controls. However, VL-17A cells exposed to 25 mM ethanol or 100 µM acetaldehyde for 30 min each exhibited a 2- and 1.6-fold higher DFCDA fluorescence, respectively, over that of the untreated control (Figure 3B). Our earlier reports that proteasome inhibition in VL-17A cells depends on EtOH oxidation corroborates these findings [23].

We tested whether acetaldehyde (Ach) generated by extracellular alcohol dehydrogenase (ADH) is associated with aggregate/aggresome formation during exposure of ^ADH−CYP2E1−^ Hep G2 cells to 50 mM EtOH. These cells were incubated with 50 mM of EtOH in the presence or absence of yeast alcohol dehydrogenase (ADH; 10 mUnits/flask) and 3 mM NAD, which were both included in the culture medium to generate Ach. Figure 3C shows that after 12 h of direct exposure of HepG2 cells to 100 μM or 300 μM Ach, or to 50 mM EtOH alone, there was no Ach detected in the medium and no evidence of toxicity, as determined by extracellular LDH activity in the culture medium. The latter exposures caused only a slight increase in intracellular aggresomes (Figure 3D). In contrast, when we exposed HepG2 cells to 50 mM EtOH in the presence of extracellular ADH and NAD^+^, we detected 500 uM Ach in the medium and a 4-fold rise in extracellular LDH activity compared with unexposed HepG2 cells (Figure 3C). The latter results were associated with a 25-fold rise in aggresome puncta numbers compared with unexposed HepG2 cells (Figure 3D), showing a clear association between Ach generation and aggresome formation. One of the reasons that direct exposure of acetaldehyde to HepG2 cells did not induce protein aggregation is because direct addition of acetaldehyde to incubation media resulted in complete depletion of its concentration, as acetaldehyde is a highly unstable compound. On the other hand, the acetaldehyde generating system (AGS) continuously produces acetaldehyde to maintain media acetaldehyde concentration stably throughout the experiment; thus, AGS induced proteasome activity decline and caused intracellular protein aggregation.

### 3.4. Autophagy Activation Prevented Aggresome Formation

**Figure 4:**
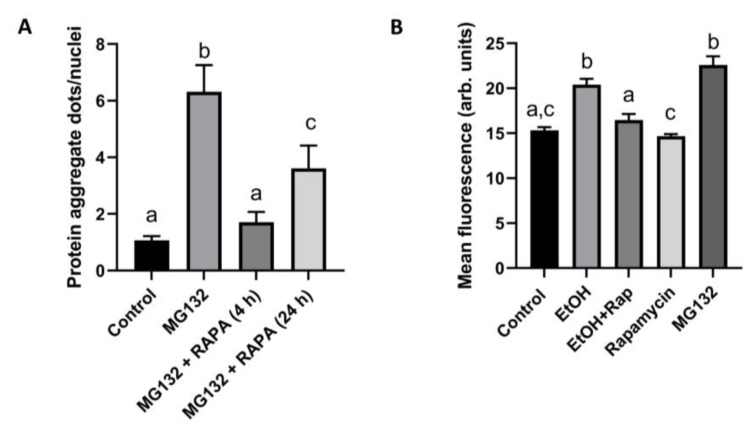
(**A**) Protein aggregates in VL-17A cells treated with MG132 for 16 h and then exposed to 100 nM rapamycin as indicated in the abscissa and (**B**) flow cytometric analysis of aggresome fluorescence in VL-17A cells treated with 25 mM EtOH for 48 h and then exposed to 100 nM rapamycin for 4 h. Some cells were treated with MG132 for 16 h. Letters have same meaning as in Figure 1 and Figure 2.

We ascertained whether autophagy activation reversed aggresome accumulation by incubating VL-17A cells overnight (16 h) with either zero or 2.5 uM MG132. We then exposed the cells to zero or 100 nM rapamycin for 4 h to activate autophagy. These analyses revealed that rapamycin exposure essentially normalized aggregate numbers to levels that equaled those of unexposed control cells. Interestingly, when the cells were treated with rapamycin for 24 h, aggresome numbers were significantly higher than in 4 h rapamycin-treated cells, but the number of 24 h aggregates in cells was still significantly lower than in cells treated with the proteasome inhibitor alone (Figure 4A). To establish whether autophagy activation blocks EtOH-induced aggresome formation, we quantified aggresome fluorescence by flow cytometry after exposing VL-17A cells to 25 mM EtOH for 48 h and then exposing them for 4 h to 100 nM rapamycin (with or without 25 mM EtOH). Overnight (16 h) exposure of VL-17A cells to 25 mM EtOH or to 2.5 uM MG132 each increased aggresome fluorescence by 1.4-fold over unexposed cells (Figure 4B). Rapamycin treatment of EtOH-exposed cells completely prevented EtOH-induced aggresome fluorescence. Cells treated with rapamycin alone exhibited basal fluorescence similar to that in untreated control cells (Figure 4B).

## 4. Discussion

Here, we provide evidence that acetaldehyde, the primary oxidation product of EtOH oxidation in liver cells, inhibits proteasome activity to induce aggresome accumulation in EtOH metabolizing hepatoma cells. We also demonstrate that aggresomes may contribute to cytotoxicity but that autophagy activation by rapamycin attenuates EtOH-induced aggresome formation in liver cells, most likely by activating autophagy to enhance intracellular aggregate removal.

Aggresome accumulation in livers of AH patients (Figure 1) suggests that aggresomes represent a histopathological and clinical outcome. We found that 24 h of EtOH exposure to ethanol-metabolizing VL-17A cells and chronic EtOH feeding to rats both induced detectable intracellular aggresomes in the cultured cells and rat hepatocytes, respectively. In both instances, aggresome accumulation was associated with a decline in proteasome activity. Notably, inhibition of ADH activity in VL-17A cells with 4-MP, which blocks the production of acetaldehyde/ethanol metabolism, completely blocked ethanol-induced proteasome activity decline. This suggests that acetaldehyde production and/or ethanol metabolism induces the downregulation of proteasome activity. Since ethanol metabolism via CYP2E1 produces ROS in addition to acetaldehyde, and both ethanol and acetaldehyde exposure for 30 min induces ROS production in VL-17A cells, ROS may also contribute to proteasome activity decline. However, since ROS production is not as continuous as acetaldehyde, which was detected even after 24 h, we postulate that acetaldehyde is largely responsible for proteasome inhibition and ROS may play a secondary role in ethanol metabolism-dependent proteasome activity decline. Despite the relative contribution to proteasome inhibition by acetaldehyde and ROS, our overall findings suggest that aggresome accumulation is likely one of the earliest cellular responses to EtOH exposure and that such accumulation likely occurs in liver cells due to EtOH-oxidation inhibition of hepatic proteasome activity. The latter postulate is supported by our observation that selective inhibition of proteasome activity with MG132 also induces aggresome formation in liver cells. Thus, our collective findings indicate that EtOH-metabolism inhibition of proteasome activity contributes to aggresome accumulation in liver cells and that such accumulation over an extended period likely results in a pathological outcome.

Another finding that supports the latter contention is that when EtOH non-metabolizing HepG2 cells were exposed directly to acetaldehyde via an acetaldehyde-generating system [25], they also exhibited robust aggresome formation. Thus, it is likely that metabolically generated acetaldehyde initiates aggresome formation. Moreover, acetaldehyde likely inhibited proteasome activity, thereby allowing intracellular aggresome formation and accumulation. Our findings, that hepatocytes from EtOH-fed rats produced higher acetaldehyde levels, exhibited a greater decline in proteasome activity, and had higher aggresome contents, corroborate previous reports [22,26,27,28]. Our findings also confirm that acetaldehyde-elicited inhibition of proteasome activity leads to cellular aggresome accumulation. In HepG2 (EtOH non-metabolizing) cells exposed to acetaldehyde and in hepatocytes isolated from EtOH-fed rats (that endogenously produced acetaldehyde), there was cellular necrosis, as evidenced by leakage of LDH into the extracellular medium, indicating that acetaldehyde exposure likely induced cellular necrosis. While this finding revealed a clear association between acetaldehyde generation and cellular necrosis, we postulate that acetaldehyde inhibition of proteasome activity and concomitant expansion of aggresomes in the cells both contributed to necrosis, as sustained proteasome inhibition and consequent intracellular accumulation of macromolecular inclusions (such as aggresomes) are cytotoxic [29,30].

Interestingly, aggresome numbers increased with the duration of ethanol exposure but were unaffected by the alcohol dose despite the finding that the degree of proteasome inhibition increased with rising ethanol doses. We hypothesize that other cellular protective mechanisms, including autophagy, probably compensated for the inability of the proteasome to degrade protein aggregates, and thus limited aggregate accumulation. While we know that EtOH metabolism decreases liver cell macroautophagy [28], we surmise that selective autophagy such as aggrephagy, the selective removal of aggresomes [31], could be active during acute EtOH exposure, as acute EtOH treatment activates hepatic autophagy [26]. Although more investigation is necessary to assess how EtOH affects aggrephagy, our findings that rapamycin treatment blocks the formation of both MG132- and EtOH-induced aggresomes indicate that autophagy activation accelerates the removal of cellular aggresomes. Collectively, these results reveal that in VL-17A cells exposed to EtOH, the degree of aggrephagy activity was insufficient to completely remove aggresomes and to protect the cells. However, rapamycin treatment maximized autophagy activation to reverse EtOH-induced aggresome formation and alleviate cellular stress.

In summary, we have demonstrated that aggresome formation is a pathophysiological outcome that is clinically relevant. While a correlation between proteasome activity decline and associated protein aggregation was previously reported in cells that metabolize ethanol solely via CYP2E1, here we used recombinant HepG2 cells that metabolize ethanol via both ADH and CYP2E1 and show that generation of the primary EtOH metabolite, acetaldehyde, during EtOH oxidation, inhibits proteasome activity. Furthermore, acetaldehyde-dependent proteasome inhibition cause aggresome accumulation inside liver cells to trigger cytotoxicity. Notably, we also show that autophagy activation removes and/or prevents the formation of aggresomes to partially or fully restore liver cell homeostasis.

## Data Availability

All data are contained within the article.

## Supplementary Materials

The following supporting information can be downloaded at: https://www.mdpi.com/article/10.3390/cells12071013/s1, Table S1: Clinical data associated with normal and AH samples.
